# Developing an Effective Peptide-Based Vaccine for COVID-19: Preliminary Studies in Mice Models

**DOI:** 10.3390/v14030449

**Published:** 2022-02-22

**Authors:** Haiqiang Yang, Jessica Cao, Xiaoyang Lin, Jingwen Yue, Tarek Zieneldien, Janice Kim, Lianchun Wang, Jianmin Fang, Ruo-Pan Huang, Yun Bai, Kevin Sneed, Chuanhai Cao

**Affiliations:** 1 Department of Pharmaceutical Sciences, Taneja College of Pharmacy, University of South Florida, Tampa, FL 33612, USA; haiqiangyang@usf.edu (H.Y.); xlin3@usf.edu (X.L.); tarekz@mail.usf.edu (T.Z.); janicekim1@usf.edu (J.K.); ksneed@usf.edu (K.S.); 2 Department of Kinesiology, Wiess School of Natural Sciences, Rice University, Houston, TX 77005, USA; jessica.a.cao@rice.edu; 3 Byrd Alzheimer’s Research Institute, Morsani College of Medicine, University of South Florida, Tampa, FL 33612, USA; yuejingwen@gmail.com (J.Y.); lianchunw@usf.edu (L.W.); 4 Department of Molecular Pharmacology and Physiology, Morsani School of Medicine, University of South Florida, Tampa, FL 33612, USA; 5 RayBiotech Life, Inc., 3607 Parkway Lane Suite 200, Peachtree Corners, GA 30092, USA; david@raybiotech.com (J.F.); rhuang@raybiotech.com (R.-P.H.); 6 MegaNanoBioTech, Inc., 3802 Spectrum Blvd. Suite 122, Tampa, FL 33612, USA; ybai@megananobiotech.com

**Keywords:** COVID-19, peptide, spike protein, vaccine, adjuvant, T and B cells, neutralizing activity

## Abstract

Coronavirus disease 2019 (COVID-19) has caused massive health and economic disasters worldwide. Although several vaccines have effectively slowed the spread of the virus, their long-term protection and effectiveness against viral variants are still uncertain. To address these potential shortcomings, this study proposes a peptide-based vaccine to prevent COVID-19. A total of 15 B cell epitopes of the wild-type severe acute respiratory coronavirus 2 (SARS-CoV-2) spike (S) protein were selected, and their HLA affinities predicted in silico. Peptides were divided into two groups and tested in C57BL/6 mice with either QS21 or Al(OH)_3_ as the adjuvant. Our results demonstrated that the peptide-based vaccine stimulated high and durable antibody responses in mice, with the T and B cell responses differing based on the type of adjuvant employed. Using epitope mapping, we showed that our peptide-based vaccine produced antibody patterns similar to those in COVID-19 convalescent individuals. Moreover, plasma from vaccinated mice and recovered COVID-19 humans had the same neutralizing activity when tested with a pseudo particle assay. Our data indicate that this adjuvant peptide-based vaccine can generate sustainable and effective B and T cell responses. Thus, we believe that our peptide-based vaccine can be a safe and effective vaccine against COVID-19, particularly because of the flexibility of including new peptides to prevent emerging SARS-CoV-2 variants and avoiding unwanted autoimmune responses.

## 1. Introduction

Coronavirus disease 2019 (COVID-19) is caused by the severe acute respiratory coronavirus 2 (SARS-CoV-2), which is a single-strand positive RNA virus that enables the host cell to directly synthesize proteins upon viral entry and uncoating [1]. This enables viral replication and the rate of viral spread to occur much more rapidly than other types of viruses [2]. Given this alarming rate of transmission, many countries were unable to implement an effective and timely approach to curb the spread of the virus, and the worldwide economic impacts of COVID-19 have been historically devastating [3].

Other viral outbreaks, such as SARS-CoV and Middle East respiratory syndrome coronavirus (MERS-CoV), have led to the development of several treatment strategies [4,5,6]. Drugs such as Nafamostat, Camostat, Chloroquine, Imatinib, Hydroxychloroquine, Ivermectin, and Remdesivir have either been studied, evaluated, or have received limited approvals by the U.S. Food and Drug Administration (FDA) to treat severe cases of COVID-19 [4,7]. Infected patients have also been treated with convalescent sera, purified antibodies from sera, or generated monoclonal antibodies from patients [8,9]. While these methods are effective, they are not viable large-scale solutions, since resources for convalescent sera therapy are limited, and producing monoclonal antibodies is time consuming and expensive [9,10,11,12,13,14].

The most effective approach to combat infectious disease is through prevention using vaccines. Vaccines have led to the eradication of smallpox, the near-elimination of poliomyelitis, and the prevention of hepatitis B (HBV) and human papillomavirus (HPV) [15,16,17,18,19]. There are four general vaccine types for preventing viral infections: (I) “classic” vaccines that utilize inactivated or attenuated whole virus [20,21], (II) nucleic acid vaccines (DNA and RNA) that elicit the host to produce antigenic fragments of the virus [22], (III) subunit vaccines that use recombinant proteins or synthetic peptides representing antigenic fragments of the virus [22], and (IV) viral vector-based vaccines that utilize viral vectors to deliver genetic material to host cells [20,23]. COVID-19 vaccines that have been approved by the U.S. FDA include classic inactivated viral particle vaccines, RNA-based vaccines, subunit-based (recombinant S protein) vaccines, and viral vector-delivered vaccines [24,25,26,27,28,29,30,31,32,33].

Coronaviruses enter host cells by binding to a cell surface receptor for viral attachment, then entering endosomes, and eventually fusing viral and lysosomal membranes [34]. This entry process is mediated by a virus surface-anchored spike (S) protein [35]. For mature viruses, the S protein is present as a trimer, with three receptor-binding S1 heads sitting on top of a trimeric membrane fusion S2 stalk [36]. The receptor-binding domain (RBD) on S1 specifically recognizes angiotensin-converting enzyme 2 (ACE2) as its receptor [37]. The SARS-CoV-2 spike protein needs to be proteolytically activated at the S1/S2 boundary to fuse membranes, such that S1 dissociates and S2 undergoes a dramatic structural change [38]. These SARS-CoV-2 entry-activating proteases include cell surface protease TMPRSS2 and lysosomal proteases cathepsins [39]. In general, the RBD domain on the S protein of SARS-CoV-2 serves as a ligand for ACE2, and ACE2 is broadly found throughout the human body [40]. Consequently, SARS-CoV-2 can quickly spread throughout the human body through binding to the ACE2 receptor [41].

COVID-19 continues to have devastating effects on public health, particularly through the occurrence of viral variants [42,43,44,45]. Some individuals who have completed the vaccine series (i.e., two doses and a booster) are still at risk of contracting COVID-19 through breakthrough infections, and those who have caught COVID-19 can also become re-infected by SARS-CoV-2 [46,47]. These infections are caused, in part, by the lack of herd immunity, but primarily because of viral mutations. Significantly decreased neutralizing potency has also been observed from the vaccines against B.1.1.7 isolate (2-fold), E484K/N501Y/D614G recombinant variant (four-fold), and two chimeric SARS-CoV-2 strains encoding B.1.351 spike (10-fold) and P.1 spike (2.2-fold) compared to the D614G variant in Vero-hACE2-TMPRSS2 cells [48]. With breakthrough infections and re-infections becoming more common, current vaccines are facing a considerable challenge in preventing the spread of COVID-19.

Understanding the advantages and disadvantages of each vaccine strategy is imperative in developing a safe and effective COVID-19 vaccine. Effective vaccines for infectious diseases ultimately require both T and B cell responses; an adjuvant is also required to prime the nonspecific immune system and ensure the robustness and duration of an immune response [49,50]. However, a vaccine with an adjuvant often might not be suitable for those with impaired immune systems (e.g., the elderly), since adjuvants can over prime the immune system [51,52]. Furthermore, age can impair immunity, so older individuals may exhibit anergy in the immune response. COVID-19 has also caused higher death rates among older populations, so a vaccine that can protect this population is crucial. Thus, each vaccine should be designed and tailored for its intended population (i.e., “personalized medicine”). As such, aged mice were utilized to provide a more thorough understanding of how the vaccine adjuvants may impact impaired immune systems.

While DNA vaccines can elicit a good T cell response, the B cell response is relatively low compared to protein-based vaccines [53,54,55,56]. Alternatively, mRNA-based vaccines are a novel approach for infectious diseases and are currently the dominant approach in fighting COVID-19 [57]. However, it remains unclear as to how effective they are in stimulating T cell and B cell responses in comparison to a more traditional protein-based vaccine or to natural infection.

Here, we developed a peptide-based vaccine for COVID-19 prevention. There are four primary advantages of peptide-based vaccines: (1) large-scale peptide synthesis is relatively inexpensive and the technology is well-established, (2) peptides can fold into three-dimensional epitopes capable of inducing antibody responses to linear and conformational structures, (3) unique epitopes can be selected to avoid autoimmune responses that could be generated by the whole protein, and (4) new epitopes can be easily added to the peptide mixture as new viral mutations are identified [58,59,60].

## 2. Materials and Methods

### 2.1. Antigen and Cell Selection

The SARS-CoV-2 spike (S) protein of the wild-type “Wuhan” strain (Accession: YP_009724390.1) was submitted to DNAStar (Madison, WI, USA) for in silico antigen (peptide) identification. Nine epitopes were selected from the S protein’s subunit 1 (S1) domain, and six sequences were selected from the subunit 2 (S2) domain. The selected peptide sequences were submitted to GenBank for homology analysis, which confirmed that the peptides were unique. Peptide antigenicity was confirmed by DNAStar.

Human Leukocyte Antigen-I (HLA-I), Mouse Major Histocompatibility Complex-I (MHC-I), and HLA-II affinity analyses were conducted with the T Cell Epitope Prediction Tools, using the Immune Epitope Database (IEBD) Analysis Resource at http://tools.iedb.org/main/tcell/, accessed on 21 July 2021. The specificity of the peptides to SARS-CoV-2 were confirmed using the protein BLAST module at https://blast.ncbi.nlm.nih.gov/Blast.cgi, accessed on 21 July 2021. All peptides were provided by Biomer Technology Inc. (Pleasanton, CA, USA).

### 2.2. SARS-CoV-2 Pseudovirus System Selection

The SARS-CoV-2 pseudovirus system was provided by Dr. Lianchun Wang’s lab at USF. A SARS-CoV-2 spike open reading frame (ORF) mammalian expression plasmid was obtained from Addgene (Plasmid #145032; Watertown, MA, USA). NL4-3 mCherry Luciferase, a replication-defective HIV dual reporter vector expressing mCherry and firefly luciferase, was obtained from Addgene (Plasmid #44965). Lipofectamine™ 3000 Transfection Reagent was purchased from Invitrogen (Waltham, MA, USA). The human 293T embryonic kidney cell line was obtained from the American-Type Culture Collection (ATCC) (Manassas, VA, USA). The 293T-ACE2-GFP cell line was established by transfecting 293T cells with the ACE2-GFP expression vector (OriGene; Rockville, MD, USA). Both cell lines were maintained in Dulbecco’s modified Eagle’s medium (DMEM) with 2 mM L-glutamine, 10% FBS, 100 μg/mL streptomycin, and 100 IU/mL penicillin. Lentiviral vector particles concentration reagent Lenti-X™ Concentrator was purchased from TaKaRa (Mountain View, CA, USA). The Luciferase Assay System was purchased from Promega (Madison, WI, USA).

For SARS-CoV-2 pseudovirus production, 4 × 10^6^ 293T cells were seeded in a 100 mm cell culture dish. After 48 h, 10 µg NL4-3 mCherry Luciferase plasmid and 10 µg SARS-CoV-2 spike plasmid were mixed in 1.5 mL Opti-MEM medium and incubated at RT for 5 min. Then, 65 μL of Lipo3000 was mixed in 1.5 mL Opti-MEM medium at RT for 5 min, and the plasmid mixture was then mixed with the Lipo3000 mixture and incubated at RT for 20 min. The mixture was added dropwise into a dish and incubated in the cell culture incubator. After 48 h, cells were centrifuged at 500× *g* for 10 min, cell supernatant was collected, and 9 mL of supernatant was transferred into a separate tube with a 3 mL Lenti-X concentrator. The supernatant was stored at 4 °C for 4 h and centrifuged at 1500× *g* for 45 min. The supernatant was aspirated, and the precipitate was resuspended with 90 μL cold DPBS and then frozen at −80 °C.

### 2.3. Mice

C57BL/6 mice were used for this study. The mice were 6 months old, gender balanced in each group, and housed at the animal facility at the University of South Florida (USF) Health Center. All animal experiments were approved (IS00008370) by the Institutional Animal Care and Use Committee (IACUC) at USF and performed according to the National Institute of Health (NIH) guidelines.

### 2.4. Vaccine Preparation

The peptides were split into two groups by S protein subdomain; each group contained peptides with high affinity to MHC (HLA) class I and class II to ensure that the peptide mixture would cover most MHC typing. HLA-I was predicted against all available allele types, and the top 1% was considered high affinity. HLA-II was also tested against all allele types in the database, and the selected standard was set at 10% rank as recommended.

A total of 15 peptides were identified against the S protein. Each peptide was dissolved with DMSO at 50 mg/mL, grouped into two groups, and mixed at 50 µg each. The two most commonly utilized adjuvants—QS21 and Al(OH)_3_ (0.4% Alhydrogel from InvivoGen (Cat no# vac-alu-50, San Diego, CA, USA)—were selected for the vaccine formula. QS21 was purchased from Desert King International (San Diego, CA, USA), and Al(OH)_3_ was purchased from Invitrogen (Waltham, MA, USA). Common adjuvants include Al(OH)_3_, which can promote earlier antibody production, and QS21, which promotes the release of Th1 cytokines participating in the elimination of intracellular pathogens [61,62,63]. The concentration of adjuvant is 25 µg per dose.

### 2.5. Vaccination and Sample Collection

Two total studies were conducted in this report, with peptide mixture groups 1 and 2 using different groups of mice. Group 1 mice (C57BL/6) were 6 months old and injected with A1-G1 (8 peptides); group 2 mice were 8 months old and received H1-E2 (7 peptides) at equal mixture amounts (50 μg/peptide) with either QS21 or Al(OH)_3_ as the adjuvant via bi-weekly subcutaneous injections for 6 injections.

**Scheme 1:**
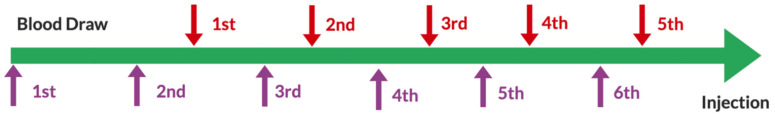
Time schedule of vaccination and blood collection for the C57BL6 mice.

Blood samples were obtained with EDTA vacutainer tubes 7 days after each injection, starting from the 2nd injection. Additionally, 50 μL of whole blood was taken for flow cytometry analysis and the rest of the blood was centrifuged at 300× *g* for 5 min. Plasma was then transferred into a new tube and stored at −80 °C for future application. The immunization and blood drawing schedule was conducted as shown in Scheme 1.

### 2.6. Indirect ELISA

The SARS-CoV-2 S protein’s full length S1 (Cat#230-30161) and receptor binding domain (RBD) (Cat#230-30162) recombinant proteins expressed in human HEK293 cells were purchased from RayBiotech Life, Inc. (Peachtree Corners, GA, USA). The SARS-CoV-2 S protein’s S1 (Cat#230-01101) and S2 (Cat#230-01103) subunit recombinant proteins expressed in *E. coli* were also purchased from RayBiotech Life, Inc.

Immulon 4 HBX 96-well plates (Cat# 3855) were purchased from Thermo Fisher Scientific (Waltham, MA, USA). HRP conjugated anti-Mouse IgG antibodies were ordered from SouthernBiotech (Birmingham, AL, USA; cat no. 1030-05), and HRP-conjugated anti-Human IgG antibodies were ordered from SouthernBiotech (Cat: 2015-05). Human plasma was purchased from RayBiotech Life, Inc.

An indirect ELISA was conducted to measure antibody response. First, 96-well plates were coated with 50 μL of peptides from 10 µg/mL S1 and S2 and 1 µg/mL recombinant protein. The plates were washed 4 times with PBST, 180 μL/well of 0.05% BSA buffer was added, and the plates were incubated at 37 °C for 1 h. The plates were then washed with 1× wash buffer, and diluted plasma was added to each well at 1:200, 1:800, and 1:3200-fold dilutions and incubated for 1 h at room temperature (RT). Anti-human or anti-mouse HRP-conjugated antibodies were then added at 1:10,000 dilutions, incubated for 1 h at RT, and washed with 1× wash buffer. Then, 100 μL of TMB substrate was added, followed by 0.4 M sulfuric acid to stop the reaction. Plates were read at OD450, with OD650 as the reference.

For epitope mapping of mouse and human plasma, plasma was added to the antigen-coated 96-well ELISA plate for indirect ELISA as described above at 1:200, 1:800, and 1:3200-fold dilutions and incubated for 1 h at RT.

### 2.7. T Cell Detection

T cell population detection post vaccination was assessed using flow cytometry assay and 50 μL whole EDTA blood. Red blood cells were lysed with ACK buffer and washed twice with 1% (*v*/*v*) FBS in 1× PBS. The fluorophore-conjugated antibody mixture was added to the sample and incubated on ice for 20 min. FITC anti-mouse CD3ε Antibody (Cat#10036), APC/Cyanine7 anti-mouse CD8a Antibody (Cat#100714), APC anti-mouse CD4 Antibody (Cat#100412), and PE anti-mouse CD19 Antibody (Cat#115508) were bought from Biolegend (San Diego, CA, USA). Cells were then resuspended with 1% FBS in 1× PBS after washing 3 times. All samples were run on the flow cytometer.

### 2.8. Neutralization Assay

ACE2-293T-GFP cells were seeded into a poly-lysine-coated 96-well plate at a density of 1.2 × 10^4^ cells/well. After 24 h, 6 μL of SARS-CoV-2 pseudovirus and diluted human plasma or mouse samples were added at the designated dilutions, and the medium was changed after 6 h of incubation. The plates were incubated in a CO_2_ incubator for 48 h, the medium was removed, and the cells were washed with Hank’s balanced salt solution (HBSS). HBSS was aspirated and 20 μL of Promega lysis buffer was added at RT for 5 min. Luminescence was measured after adding 50 μL of Luciferase substrate into 20 μL of lysis buffer.

### 2.9. Immunoglobulin Isotyping

The mouse immunoglobulin isotyping (Cat#MGAMMAG-300K) and cytokine magnetic bead panel assay (Cat#MCYTOMAG-70K) were purchased from MilliporeSigma (Burlington, MA, USA).

First, 100 μL of assay buffer was added to each well of the assay plate and mixed on a plate shaker for 10 min at RT. The assay buffer was then discarded, and the remaining residue was removed from the wells by inverting the plate and tapping it numerous times on an absorbent towel. Then, 50 μL of the assay buffer was added to the background wells, and 50 μL of the standard, control, and diluted samples were added to the appropriate wells. Afterwards, the MILLIPLEX MAP Anti-Mouse Multi-Immunoglobulin beads were vortexed at medium speed for 15 s and sonicated for 15 s using a sonication bath. Then, 25 μL of the bead solution was added into each well, and the plate was covered and incubated on a plate shaker for 15 min at RT. The plate was then washed twice with 200 μL/well of washing buffer. After washing, 25 μL/well of prepared Anti-Mouse κ Light Chain PE was added, covered, and incubated on a plate shaker at RT for 15 min. Finally, the fluid was aspirated, and beads were resuspended in 150 μL/well of sheath fluid. The plate was then read on the Bio-Rad Bio-Plex MAGPIX Multiplex Reader.

### 2.10. Cytokine Detection with Multiplex Assay

First, 200 μL of washing buffer was added to each well, and the plate was sealed and incubated on the plate shaker for 10 min at RT. The wash buffer was then aspirated, and 25 μL of each standard or control was introduced into the appropriate wells, with the assay buffer utilized for the 0 pg/mL standard (background). Then, 25 μL of the assay buffer was added to the sample wells, and 25 μL of the serum matrix solution was added to the background, standard, and control wells. Afterwards, 25 μL of the diluted samples were introduced into the appropriate wells. Then, the mixing bottle was vortexed, and 25 μL of the mixed or premixed beads were added to each well and shaken for 10 min at RT. The plate was then sealed with a plate sealer, wrapped with aluminum foil, and incubated on a plate shaker overnight at 2–8 °C. The following day, well contents were gently removed, and the plate was washed twice. Then, 25 μL of the detection antibodies were added into each well and incubated on a plate shaker for 1 h at RT. The well contents were removed, the plates were washed twice, and 150 μL of sheath fluid was added to all wells. The beads were then resuspended on a plate shaker for 5 min, and the plate was read on the Bio-Rad Bio-Plex MAGPIX Multiplex Reader.

## 3. Results

### 3.1. Selection of SARS-CoV-2 Peptides

Sixteen peptides were predicted to have high antigenicity with 100% homology to the SARS-CoV-2 virus were split across two peptide mixtures (Group 1, Group 2) that covered all Class I and II allele types of C57B/6 mice. The peptide sequences and T cell epitope results are listed in Table 1 and Supplementary Table S1, respectively.

### 3.2. Antibody Response Post Vaccination Using Group 1 Peptides with Different Adjuvants

**Figure 1:**
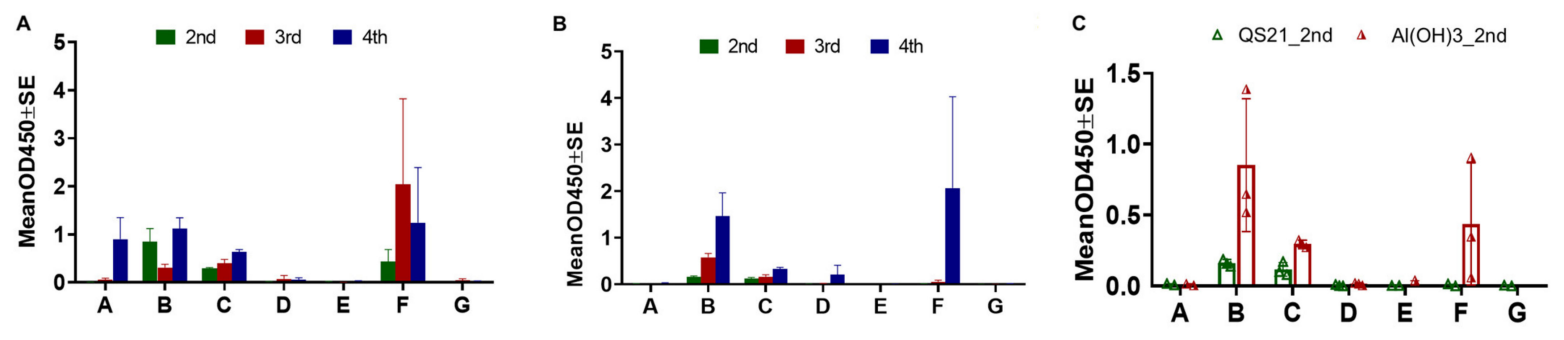
Antibody response post-vaccination tested by ELISA. Test subjects (mice) were divided into 2 groups (n = 3 per group): one group received the peptide mixture 1 (mainly S1) with QS21 as the adjuvant, and the other group received the peptide mixture 1 with Al(OH)_3_ as the adjuvant. There were a total of 6 inoculations at 2-week intervals and 5 time points where blood samples were collected. Plasma was analyzed by ELISA with peptide A1-G1-coated plates. In total, 3 time points were selected for comparison. (**A**) shows the results of the 3 mice that received the Al(OH)_3_ adjuvant, (**B**) the results of the three mice with QS21 as adjuvant, and (**C**) is the comparison of blood post 2nd vaccination. There are significant antibodies in both adjuvant groups against peptides B (*p* = 0.0058 < 0.01) and F (*p* = 0.0009 < 0.01) (**C**). No antibodies were induced against peptides A, D, E, or G after 2 vaccinations.

First, 50 ug/peptide of peptides A–G of the S1 protein were mixed with either the QS21 or Al(OH)_3_ adjuvant and injected into mice subcutaneously at two-week intervals. Blood was collected 10 days after each injection and used for antibody detection. It is evident that, after two injections, both groups could detect an antibody response, but the Al(OH)_3_ group exhibited a higher antibody response than the QS21 group. With continuous boosting, antibody response to each peptide differed between the two adjuvants (Figure 1).

### 3.3. Antibody Response to Group 2 Peptides with Different Adjuvants

**Figure 2:**
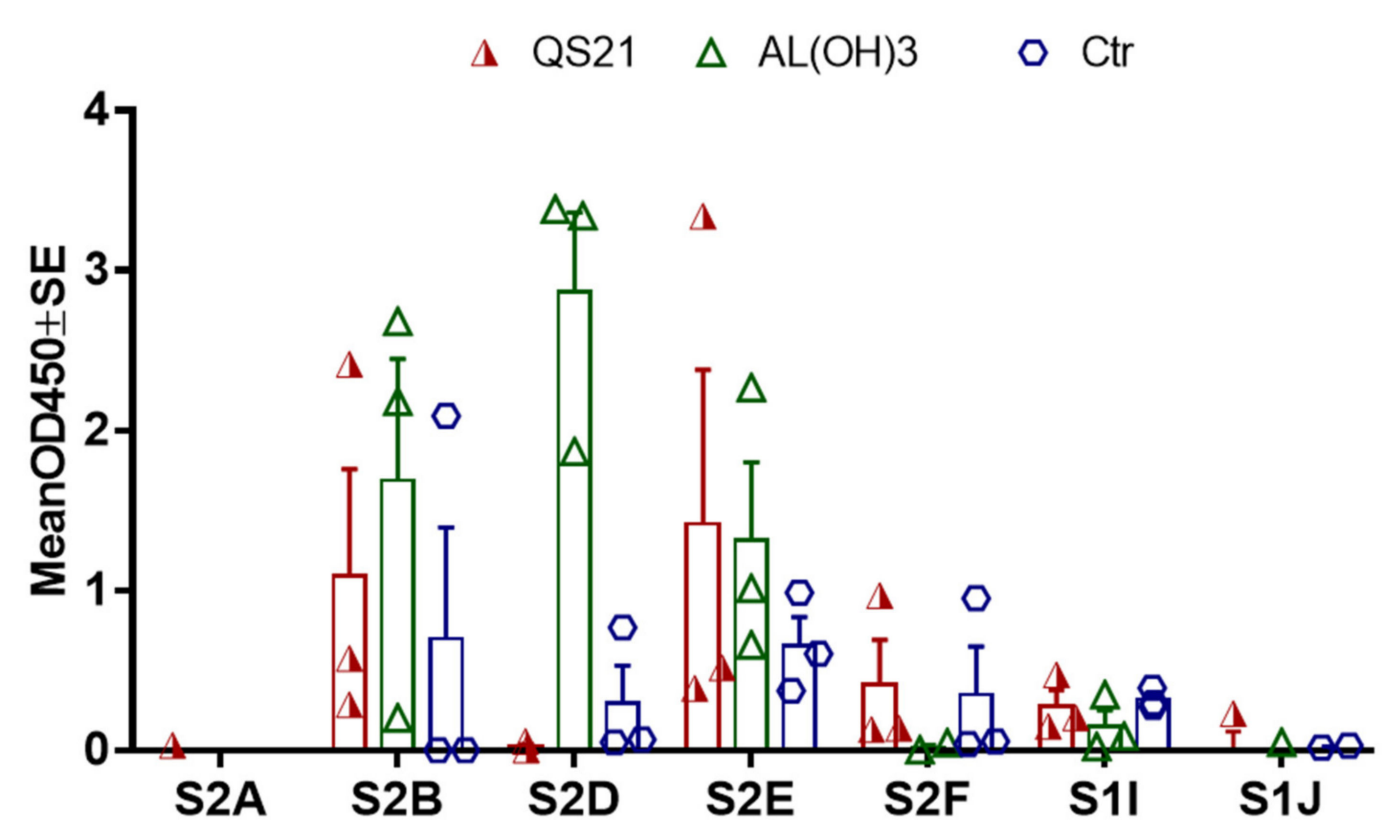
Antibody response to peptide mixture after 2 vaccines. In total, 7 peptides were used in this injection, with either QS21 or Al(OH)_3_ as adjuvants. Peptides S1I and S1J are selected from S1 domain. Peptide S2A, S2B, S2D, S2E and S2F are selected from S2 domain. (N = 3, *p* < 0.001).

After the Group 1 peptide test, another group of peptides were injected into mice, with either QS21 or Al(OH)_3_ adjuvants. There were a total seven peptides in this group—two in the S1 domain and five in the S2 domain. The ELISA results showed that antibodies were produced against five peptides in both QS21 and Al(OH)_3_ groups but were relatively lower in the QS21 group than the Al(OH)_3_ adjuvant group; Al(OH)_3_-2D produce higher antibodies than the QS21 adjuvant group (*p* < 0.001). No antibodies were induced against peptide 2A and 1J after two injections (Figure 2).

### 3.4. The Similarity of Epitope Mapping between Recovered Human Sera and Vaccinated Mouse Sera

**Figure 3:**
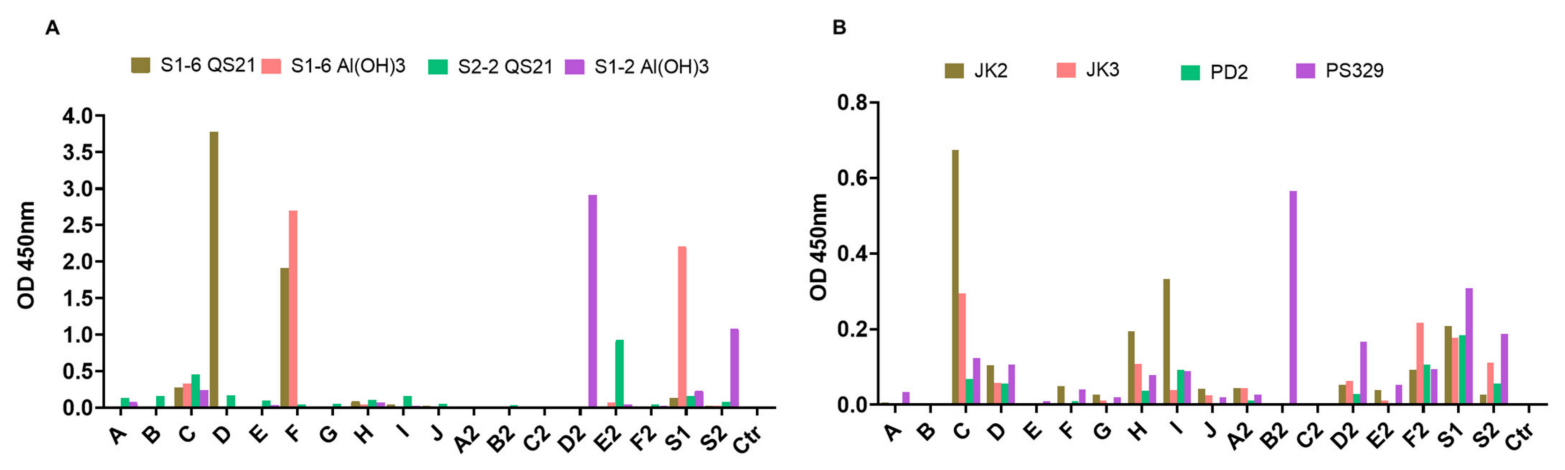
Epitope mapping results of vaccinated mouse plasma (**A**) versus human recovered plasma (**B**). Coating peptides A–J are peptides from the S1 protein, A2–F2 are peptides from the S2 protein. S1 is the recombinant S1 of the S protein and S2 is the recombinant S2 of the S protein. Ctr is a control peptide of human alpha-synuclein protein, which served as a negative control. Plasma was diluted at a 1:800 dilution with the sample, and ELISA results were shown in the graphs.

Four mice plasma (two from each peptide mixture group) and four human plasma samples (two recovered patient blood and two from our tissue bank collected in 2008 as negative controls) were selected for epitope mapping. A total of 16 peptides and recombinant S1 and S2 proteins were predicted in this assay. JK2 and JK3 (recovered patients) display a pattern similar to our peptide vaccine, indicating that the vaccine has included all major epitopes (binding to C, D, H1, I1) (Figure 3).

### 3.5. The Duration of Antibody Response Post-Vaccination

**Figure 4:**
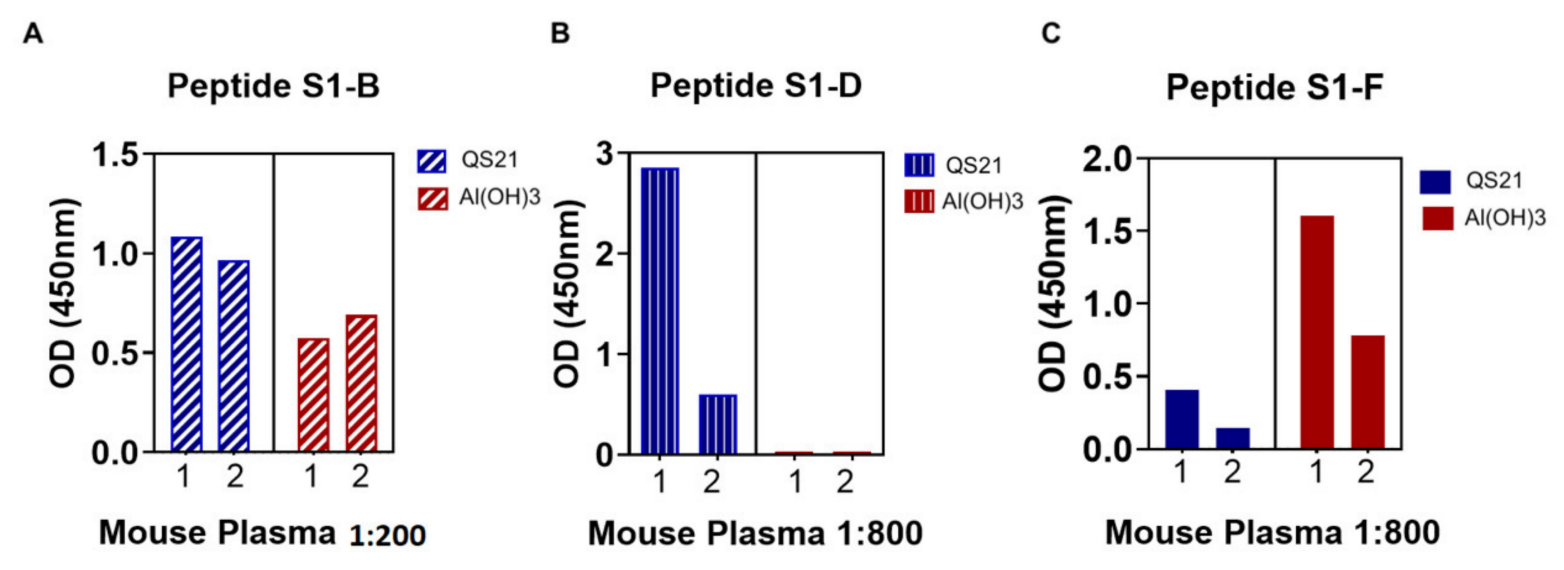
Antibody duration between two adjuvants. The graphs above are the results of antibody testing via ELISA on blood from two time points (1 and 2): 1 is 10 days after the 6th vaccination and 2 is three months after 1. Al(OH)_3_ can generate a longer antibody response than QS21 (**B**,**C**). However, QS21 can induce a higher antibody response to some antigens, such as that seen in S1-B (**A**).

When examining the duration of the antibody response post-vaccination, Al(OH)_3_ appears to generate a longer antibody response than the QS21 adjuvant. Nonetheless, QS21 induced a higher antibody response to some antigens, such as the ones seen in peptide S1-B (Figure 4). 

### 3.6. Cell Population Analysis to Different Adjuvant Vaccines

**Figure 5:**
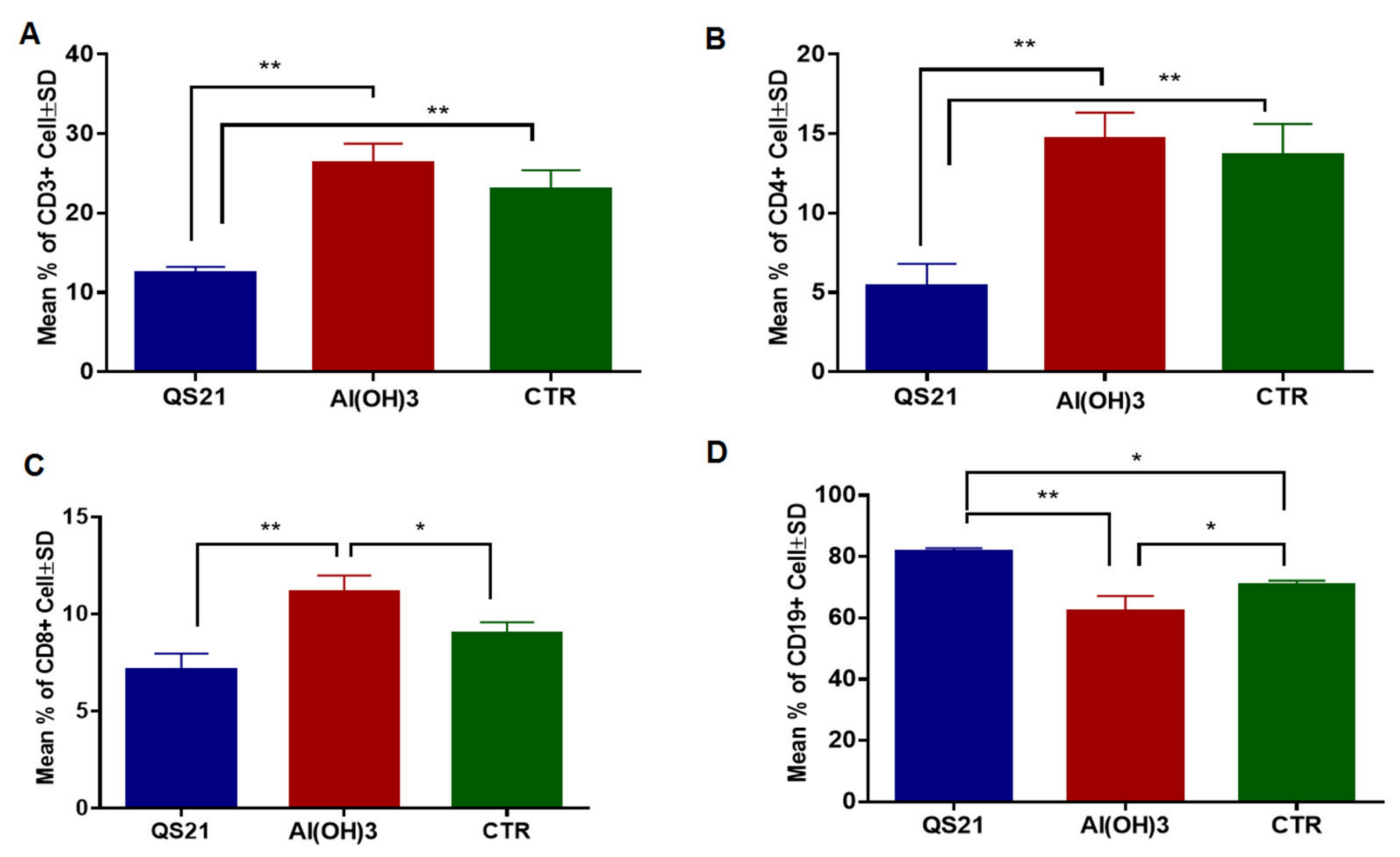
B and T cell population detection post vaccination. (**A**) CD3+ cell population; (**B**) CD4+ cell population; (**C**) CD8+ cell population; (**D**) CD19+ cell population. (* *p* < 0.05, ** *p* < 0.01, N = 3 per group).

Mice blood samples were collected after the third vaccination of peptide mixture group 1 with different adjuvants to compare the B and T cell population difference. Flow cytometry assay was used to analyze the T and B cell population for each vaccine adjuvant. CD3+ and CD8+ T cells (Figure 5A,C) were significantly higher in Al(OH)_3_ groups than QS21 groups (*p* < 0.05; N = 3 per group). CD4+ T cells (Figure 5B) were also significantly higher in the Al(OH)_3_ adjuvant groups compared to both the QS21 and control groups (*p* < 0.05; n = 3 per group). Interestingly, the B cell population in the Al(OH)_3_ adjuvant group was significantly decreased when compared to the QS21 adjuvant group (N = 3 per group, *α* = 0.05). These results imply that the Al(OH)_3_ adjuvant is ideal for obtaining the desired cellular response (Figure 5).

### 3.7. Neutralizing Activity to Pseudovirus System

**Figure 6:**
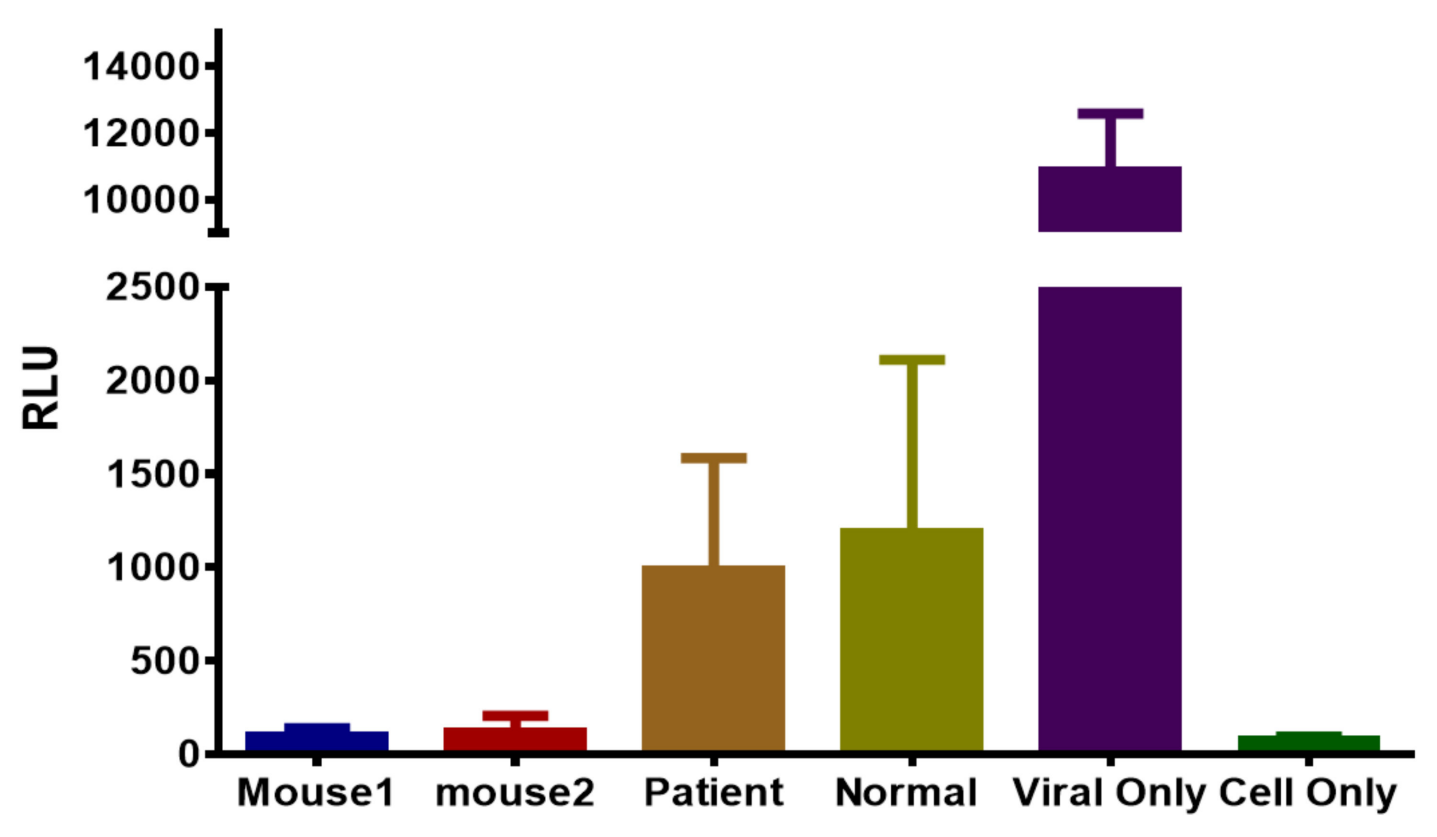
Results of neutralization assay of mouse and human plasma. Mouse1 was selected from the peptide mixture 1 group with the QS21 adjuvant; Mouse2 was from the peptide mixture 1 group with the Al(OH)_3_ adjuvant; Patient is human plasma from a recovered COVID-19 infected patient; Normal is human negative plasma; Viral Only consists of the viral infected cells without adding plasma; Neg Ctr is cell only (N = 4 per group).

The pseudovirus system in vitro was compared with recovered patient plasma to determine the neutralization potential of post-vaccinated mice plasma. The results showed that mice plasma had better neutralization function than human plasma in this system (Figure 6).

### 3.8. The Immunoglobulin Isotyping of Plasma from Vaccinated Mice

**Figure 7:**
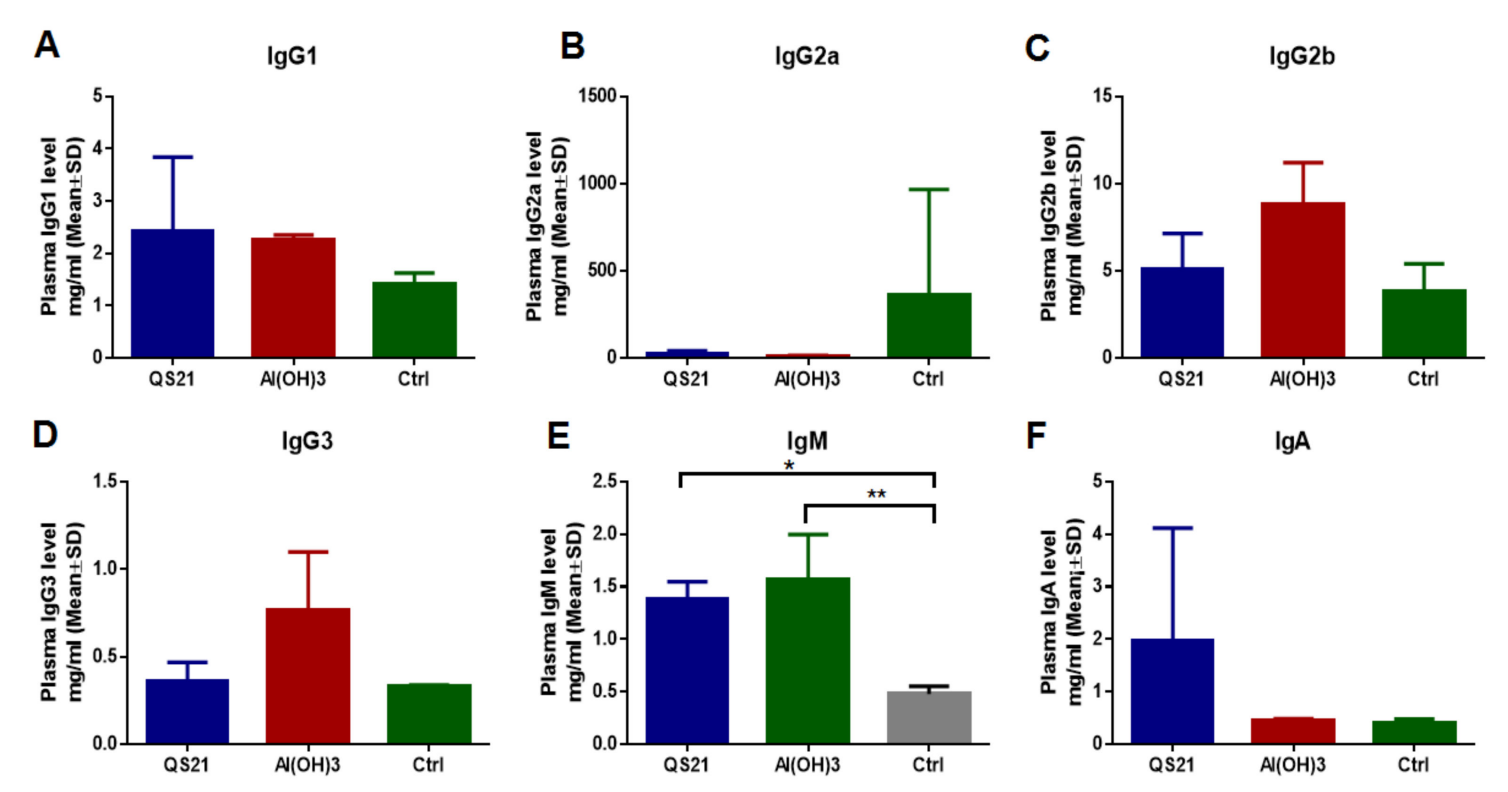
Ig Isotyping results of plasma from mice vaccinated with peptide mixture group 1. (**A**) IgG1 level; (**B**) IgG2a level; (**C**) IgG2b Level; (**D**) IgG3 Level; (**E**) IgM Level; (**F**) IgA Level; (the final dilution of mice plasma was at 1:10,000) (* *p* < 0.05, ** *p* < 0.01, N = 3 per group).

There are no differences in IgG levels among all three groups. IgM levels are higher in the vaccine group than control mice, indicating that the adjuvant-based peptide vaccine could not change the immunotype of the mice (Figure 7).

## 4. Discussion

Vaccines are undeniably the most effective solution to stopping the spread of COVID-19, and several vaccines have already been distributed to contribute to this effort. Pfizer and Moderna have developed nanolipid-based mRNA particle vaccines, while Astrazeneca and Johnson & Johnson have produced adenoviral vector-based DNA vaccines [26,64]. While these have become the most widely used vaccines to prevent COVID-19, other types of vaccines have also been developed, such as an inactivated whole viral particle vaccine (Kexing) and recombinant subunit vaccine (Novavax) [65,66,67]. Although the formulation of each vaccines varies, they all aim to block the binding of the S protein to ACE2 and inhibit viral entry into the host cell [1]. Virologists have proved that the COVID-19 virus surface has spike (S) proteins that can specifically bind to ACE2 through a receptor binding domain (RBD) [39]. The RBD on the S protein of COVID-19 serves as the ligand for ACE2 on the cell surface. It is important to note that most human organs produce the ACE2 protein [40], which may have contributed to the many deaths caused by COVID-19 that were also attributed to multiple organ failure [68].

The COVID-19 vaccines that are currently approved have been reported to have high rates of effectiveness, ranging from 66.3% to 95%, and both B and T cell responses have been observed [69,70,71,72,73,74,75,76,77,78]. Moreover, no large-scale and severe adverse effects have been reported. However, long term monitoring is still necessary before an affirmative assessment can be made regarding vaccine safety.

The human immune system works diligently to protect the body from viruses [79]. During viral infection, immune cells produce large amounts of cytokines, which can result in cytokine storm syndrome [80]. Normally, a virus has a specific tissue receptor, meaning the virus can only infect certain tissues, while causing no harm to other tissues [81]. For instance, HIV targets the CD4 receptor, only infecting and killing CD4 T cells [82,83]. However, SARS-CoV-2 is markedly different from other viruses because it utilizes ACE2 receptors, and ACE2 is widely expressed by many types of cells across different organs [40]. ACE2 bound by the SARS-CoV-2 S protein can be considered a viral particle and will trigger an immune response [84]. This scenario complicates vaccine development because the abundance of ACE2 increases the likelihood of potential adverse responses caused by the vaccine and must therefore be carefully considered.

Most of the current vaccines use the S protein as the antigen, and target for blocking viral entry by sealing RBD on S1 of COVID-19, which induces our immune system to attack cells that bind to the S protein [21]. To induce an immune response, antigen-presenting cells (APCs) must uptake the S protein, process it, and then present it to CD4 or CD8 T cells [85]. Although this process is highly effective with other vaccines, it can be problematic for S gene- or S protein-based vaccines, since the S protein will bind to the ACE2 of the host cell.

To combat the potential issues of current COVID-19 vaccines, our study uses peptides as opposed to whole S protein to develop a vaccine for COVID-19. We selected 15 peptides for the whole S protein utilizing B and T cell epitope prediction methods. We have also used two of the most common adjuvants in our vaccine formula—QS21 and Al(OH)_3_—to enhance the immune response and tested this vaccine on a mouse model. The immunization results indicate that antibody production against each peptide varies with time and adjuvant used. In the QS21 adjuvant group, peptide B was the first antibody produced, followed by antibodies against peptides C, F, and D. In the Al(OH)_3_ adjuvant group, antibodies against B, C, and F were detected at the same time after the second immunization, which was quicker than in the QS21 adjuvant group (Figure 1). Another major difference between the two adjuvants is that the Al(OH)_3_ group produced antibodies against peptide A much earlier than the QS21 group. Since it is imperative for the body to generate a sufficient T cell response to combat infectious diseases [86], the T and B cell population of each adjuvant was further analyzed. The results indicate that Al(OH)_3_ is the ideal adjuvant because it can induce a more robust T cell response than QS21 (Figure 5), although both adjuvants were able to induce an antibody response when tested using the ELISA assay. Since a high antibody titer does not always signify satisfactory protection, we also tested the protection of plasma with the pseudoviral system [87]. This showed that the plasma from the vaccinated mice can effectively neutralize viral entry (Figure 6). Another issue related to the anti-viral activity of plasma is determining how many epitopes are adequate to completely neutralize the viral entry that can be used as target sites for vaccine development [88]. To collect this information, epitope mapping with plasma from vaccinated mice and from infected patients was conducted. Epitope mapping revealed a similar antibody pattern from vaccinated mice to human samples, which confirmed that our epitope covered most B cell epitopes of the S protein (Figure 2). Thus, our results demonstrated that development of a S protein peptide-based vaccine is likely feasible to prevent COVID-19.

## 5. Conclusions

The authors show that a peptide-based COVID-19 vaccine using S protein peptides with the Al(OH)_3_ adjuvant generates a high antibody and T cell response. We also demonstrate that this peptide-based vaccine includes most major B cell epitopes of the S protein by comparing the serological antibody profiles of mice injected with our peptide-based vaccine with humans who were previously infected with COVID-19. Further investigation into peptide-based COVID-19 vaccines is warranted to evaluate important factors such as long-term efficacy.

## Figures and Tables

**Table 1:** Peptide sequence and their usage in the vaccination. A1-F2 are the peptide sequence names of each selected peptide. A cystine is added to the N-terminal of each peptide; AA position is the amino acid position in the whole S protein; vaccine group indicates the peptide mixture used for the vaccine.

Sequence Name	Peptide Sequence	AA Position in S Protein	Vaccine Group
A1	**CLPFQQFGRDIADTTDAVRDPQTLEIL**	**560–585**	Group1 (S1)
B1	**CYFKIYSKHTPINLVRDLPQ**	**200–218**	Group1 (S1)
C1	**CGVYYHKNNKSWMESEFRVY**	**142–160**	Group1 (S1)
D1	**CFHAIHVSGTNGTKRFDNPVLPF**	**65–86**	Group1 (S1)
E1	**CTRGVYYPDKVFRSSVLHS**	**33–50**	Group1 (S1)
F1	**CYQTQTNSPRRARSVAS**	**674–689**	Group1 (S1)
G1	**CVIAWNSNNLDSKVGGNY**	**443–449**	Group1 (S1)
H1	**CALDPLSETKCTLKSFTVEKGIYQTSNFRV**	**291–320**	Group2 (S1)
I1	**CATVCGPKKSTNLVKNKCVNFNFNG**	**522–545**	Group2 (S1)
J1	**CYNYLYRLFRKSNLKPFERDISTEIYQA**	**452–476**	Group2 (S1)
A2	**CIAVEQDKNTQEVFAQV**	**770–783**	Group2 (S2)
B2	**CKQIYKTPPIKDFGGFNFSQILPDPSKPSKRSFIEDLL**	**786–822**	Group2 (S2)
C2	**CNSAIGKIQDSLSSTASAL**	**927–945**	Group2 (S2)
D2	**CPLQPELDSFKEELDKYFKNHTSPDVDLGDIS**	**1141–1171**	Group2 (S2)
E2	**CVPAQEKNFTTAPAICHDGKAHFPREGVFVSNGTHWF**	**1068–1103**	Group2 (S2)
F2	**CMTSCCSCLKGCCSCGSCCKFDEDDSEPVLKGV**	**1236–1267**	Group2 (S2)

## Data Availability

The data presented in this article are available on request from the corresponding author.

## Supplementary Materials

The following supporting information can be downloaded at: https://www.mdpi.com/article/10.3390/v14030449/s1, Table S1: Selection of SARS-CoV-2 peptides.
