# Supplementary material for: Developing an Effective Peptide-Based Vaccine for COVID-19: Preliminary Studies in Mice Models
Source: Viruses. 2022 Feb 22;14(3):449. doi: 10.3390/v14030449 (PMC8954996; doi:10.3390/v14030449)
Supplement: Supplementary file 1 [file viruses-14-00449-s001.zip › viruses-1586724-supplementary.pdf]

1. Full amino acid sequence of SARS-CoV-2 spike protein (1-1273)

MFVFLVLLPLVSSQCVNLTTRTQLPPAYTNSFTRGVYYPDKVFRSSVLHSTQDLFLPFFSNVTWFHAIHVSGTNGTKRFD
NPVLPFNDGVYFASTEKSNIIRGWIFGTTLDSKTQSLLIVNNATNVVIKVCEFQFCNDPFLGVYYHKNNKSWMESEFRVY
SSANNCTFEYVSQPFLMDLEGKQGNFKNLREFVFKNIDGYFKIYSKHTPINLVRDLPQGFSALEPLVDLPIGINITRFQTLL
ALHRSYLTPGDSSSGWTAGAAAYYVGYLQPRTFLLKYNENGTITDAVDCALDPLSETKCTLKSFTVEKGIYQTSNFRVQP
TESIVRFPNITNLCPFGEVFNATRFASVYAWNRKRISNCVADYSVLYNSASFSTFKCYGVSPTKLNDLCFTNVYADSFVIR
GDEVRQIAPGQTGKIADYNYKLPDDFTGCVIAWNSNNLDSKVGGNYNYLYRLFRKSNLKPFERDISTEIYQAGSTPCNG
VEGFNCYFPLQSYGFQPTNGVGYQPYRVVVLSFELLHAPATVCGPKKSTNLVKNKCVNFNFNGLTGTGVLTESNKKFLP
FQQFGRDIADTTDAVRDPQTLEILDITPCSFGGVSVITPGTNTSNQVAVLYQDVNCTEVPVAIHADQLTPTWRVYSTGSN
VFQTRAGCLIGAEHVNNSYECDIPIGAGICASYQTQTNSPRRARSVASQSIIAYTMSLGAENSVAYSNNSIAIPTNFTISVTT
EILPVSMTKTSVDCTMYICGDSTECSNLLLQYGSFCTQLNRALTGIAVEQDKNTQEVFAQVKQIYKTPPIKDFGGFNFSQI
LPDPSKPSKRSFIEDLLFNKVTLADAGFIKQYGDCLGDIAARDLICAQKFNGLTVLPPLLTDEMIAQYTSALLAGTITSGW
TFGAGAALQIPFAMQMAYRFNGIGVTQNVLYENQKLIANQFNSAIGKIQDSLSSTASALGKLQDVVNQNAQALNTLVK
QLSSNFGAISSVLNDILSRLDKVEAEVQIDRLITGRLQSLQTYVTQQLIRAAEIRASANLAATKMSECVLGQSKRVDFCGK
GYHLMSFPQSAPHGVVFLHVTYVPAQEKNFTTAPAICHDGKAHFPREGVFVSNGTHWFVTQRNFYEPQIITTDNTFVSG
NCDVVIGIVNNTVYDPLQPELDSFKEELDKYFKNHTSPDVDLGDISGINASVVNIQKEIDRLNEVAKNLNESLIDLQELGK
YEQYIKWPWYIWLGFIAGLIAIVMVTIMLCCMTSCCSCLKGCCSCGSCCKFDEDDSEPVLKGVKLHYT

2. Antigenic prediction of COVID-19 virus spike protein

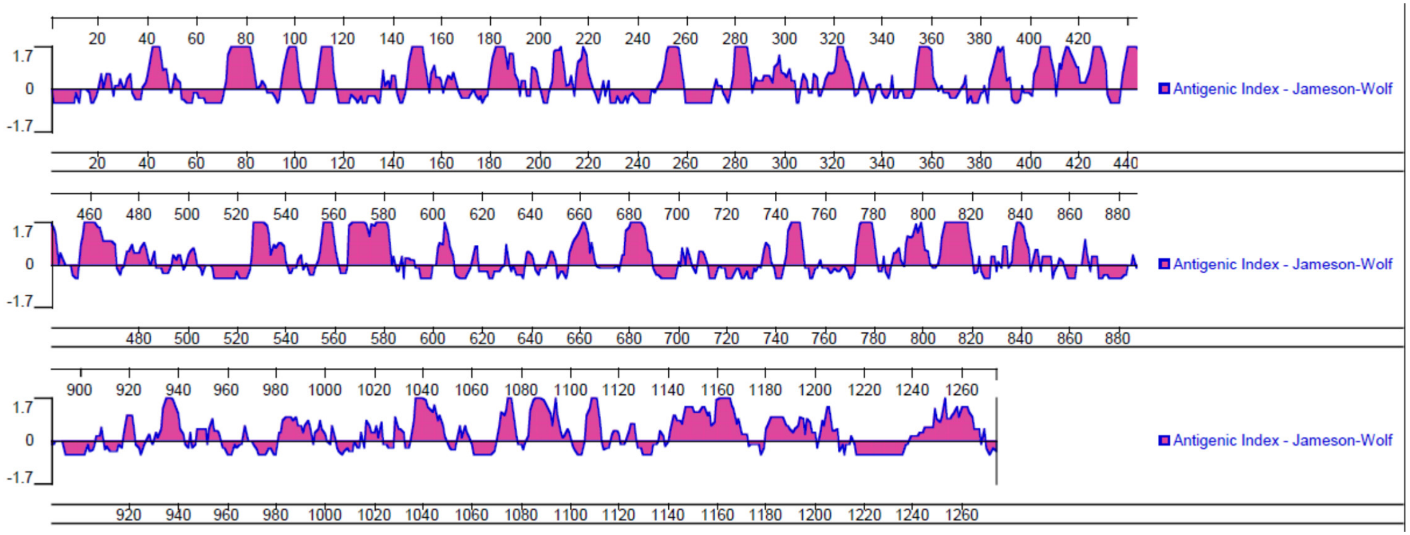

**3. Supplementary Table S1. Selection of SARS-CoV-2 peptides.** Each selected peptide binding to human HLA-I is predicted by using http://tools.iedb.org/mhci/, and all alleles are selected and binding of peptide to HLA-II is analyzed using http://tools.iedb.org/mhcii/, and peptide length is 15 amino acids against all alleles. Both peptide mixtures with QS21 or $Al(OH)_3$ induced B and T cell responses in C57BL/6 mice. Furthermore, antibody production against each individual peptide varied with injection time. Using $Al(OH)_3$ as the adjuvant, the peptide mixtures generated a long-lasting antibody response with an efficacious cellular response. These peptide mixtures, when used as a vaccine, stimulate humoral antibody production, and some peptides could be used to evaluate vaccine effectiveness.

| **Peptide name and aa sequences** | **AA position** | **Binding to MHC-I types** | **Binding to MHC-II types** | **Human HLA-II** | **Human HLA-I** |
|---|---|---|---|---|---|
| CLPFQQFGRDIADTTDAVRDPQTLEIL | 560-585 (A1) | H-2-Dd | N/A | HLA-DRB3*01:01 | HLA-A*26:01; HLA-A*30:01; HLA-A*33:01; HLA-A*68:01; HLA-A*68:02; HLA-B*08:01; HLA-B*35:01; HLA-B*51:01; HLA-B*53:01 |
| CYFKIYSKHTPINLVRDLPQ | 200-218 (B1) | H-2-Db, H-2-Dd, H-2-Kb, H-2-Kd, H-2-Ld; H-2-Qa1 | H2-IEd, H2-IAb | HLA-DPA1*02:01/DPB1*14:01; HLA-DRB1*07:01; HLA-DRB1*08:02; HLA-DRB1*09:01; HLA-DRB1*11:01; HLA-DRB1*13:02; HLA-DRB1*15:01; HLA-DRB3*02:02 | HLA-A*02:01; HLA-A*02:03; HLA-A*02:06; HLA-A*23:01; HLA-A*24:02; HLA-A*30:01; HLA-A*32:01; HLA-A*68:02; HLA-B*07:02; HLA-B*08:01; HLA-B*35:01; HLA-B*51:01; HLA-B*53:01; HLA-B*57:01;HLA-B*58:01 |
| CGVYYHKNNKSWMESEFRVY | 142-160 (C1) | H-2-Db, H-2-Kb, H-2-Kd | H2-IEd | HLA-DPA1*01:03/DPB1*04:01; HLA- | HLA-A*01:01; HLA-A*03:01; HLA-A*11:01; HLA-A*23:01; HLA-A*24:02; HLA-A*30:01; HLA-A*30:02: HLA-A*31:01; HLA-A*32:01; HLA-B*15:01; |

| | | | | | |
|---|---|---|---|---|---|
| | | | | DRB1*04:01; HLA-DRB3*02:02 | HLA-B*35:01; HLA-B*53:01; HLA-B*57:01; HLA-B*58:01 |
| CFHAIHVSGTNGTKRFDNPVLPF | 65-84 (D1) | H-2-Kb, H-2-Qa1 | H2-IEd | HLA-DRB3*02:02; HLA-DRB1*04:01; HLA-DPA1*01:03/DPB1*04:01 | HLA-A*03:01; HLA-A*11:01; HLA-A*23:01; HLA-A*24:02; HLA-A*30:01; HLA-A*30:02: HLA-A*32:01; HLA-A*33:01; HLA-A*68:01 |
| CTRGVYYPDKVFRSSVLHS | 33-50 (E1) | H-2-Dd | H2-IEd | HLA-DPA1*02:01/DPB1*05:01; HLA-DRB1*03:01; HLA-DRB1*04:01; LA-DRB1*13:02; HLA-DRB3*01:01; HLA-DRB3*02:02 | HLA-A*03:01; HLA-A*11:01; HLA-A*24:02; HLA-A*30:01; HLA-A*30:02: HLA-A*31:01; HLA-A*32:01; HLAA*33:01; HLA-A*68:01; HLA-B*07:02; HLA-B*08:01; HLA-B*15:01; HLA-B*35:01; HLA-B*51:01; HLA-B*53:01 |
| CYQTQTNSPRRARSVAS | 674-689 (F1) | H-2-Dd | N/A | HLA-DRB1*04:01 | HLA-A*03:01; HLA-A*11:01; HLA-A*30:01; HLA-A*33:01: HLA-A*68:01; HLA-B*07:02; HLA-B*08:01 |
| CVIAWNSNNLDSKVGGNY | 433-449 (G1) | H-2-Db, H-2-Kb | N/A | HLA-DRB3*02:02; HLA-DRB1*13:02 | HLA-A*01:01; HLA-A*30:02; HLA-A*68:02 |
| CALDPLSETKCTLKSFTVEKGIYQTSNFRV | 291-320 (H1) | H-2-Dd, H-2-Kb, H-2-Kd H-2-Qa1 | N/A | N/A | HLA-A*01:01; HLA-A*03:01; HLA-A*11:01; HLA-A*23:01; HLA-A*24:02; HLA-A*30:01; HLA-A*30:02: HLA-A*31:01; HLA-A*32:01; HLA-A*33:01; HLA-A*68:01; HLA-A*68:02; HLA-B*44:01; HLA-B*51:01; HLA-B*53:01; HLA-B*57:01; HLA-B*58:01 |
| CATVCGPKKSTNLVKNKCVNFNFNG | 522-545 (I1) | H-2-Db, H-2-Dd, H-2-Kb, H-2-Ld; H-2-Qa1 | N/A | LA-DRB1*13:02; HLA-DRB3*02:02 | HLA-A*03:01; HLA-A*11:01; HLA-A*30:01; HLA-A*31:01: HLA-B*07:02; HLA-B*08:01; HLA-B*15:01; HLA-B*51:01 |

| | | | | | |
|---|---|---|---|---|---|
| CYNYLYRLFRKSNLKPFERDISTEIYQA | 452-476 (J1) | H-2-Kk, H-2-Qa2 | H2-IEd | HLA-DPA1*01:03/DPB1*02:01; HLA-DPA1*01:03/DPB1*04:01; HLA-DPA1*02:01/DPB1*01:01; HLA-DPA1*02:01/DPB1*05:01; HLA-DRB1*04:01; HLA-DRB1*04:05; HLA-DRB1*07:01; HLA-DRB1*08:02; HLA-DRB1*11:01; HLA-DRB1*12:01; HLA-DRB1*15:01; HLA-DRB3*01:01; HLA-DRB3*02:02; HLA-DRB5*01:01 | HLA-A*01:01; HLA-A*03:01; HLA-A*11:01; HLA-A*23:01; HLA-A*24:02; HLA-A*30:01; HLA-A*31:01; HLA-A*33:01; HLA-A*68:02; HLA-B*07:02; HLA-B*08:01; HLA-B*15:01; HLA-B*40:01; HLA-B*44:02; HLA-B*44:03 |
| **C**IAVEQDKNTQEVFAQV | 770-783 (A2) | H-2-Db, H-2-Kb, H-2-Kk, H-2-Qa2 | N/A | N/A | HLA-A*02:01; HLA-A*02:03; HLA-A*02:06; HLA-A*26:01; HLA-A*68:02; HLA-B*15:01; HLA-B*40:01; HLA-B*44:02; HLA-B*44:03; HLA-B*51:01 |
| CKQIYKTPPIKDFGGFNFSQILPDPSKPSKRSFIEDLL | 786-822 (B2) | H-2-Db, H-2-Dd, H-2-Kb, H-2-Kd, H-2-Qa2 | H2-IAb | HLA-DPA1*01:03/DPB1*02:01; HLA-DPA1*01:03/DPB1*04:01; LA-DPA1*02:01/DPB1*01:01; HLA-DQA1*01:01/DQB1*05:01; HLA-DRB1*03:01; HLA-DRB1*04:01; HLA- | HLA-A*02:01; HLA-A*02:03; HLA-A*02:06; HLA-A*03:01; HLA-A*11:01; HLA-A*23:01; HLA-A*24:02; HLA-A*30:01; HLA-A*32:01; HLA-A*68:01; HLA-B*07:02; HLA-B*08:01; HLA-B*35:01; HLA-B*53:01; HLA-B*57:01 |

| | | | | | |
|---|---|---|---|---|---|
| | | | | DRB1*04:05; HLA-DRB1*09:01 | |
| **C**NSAIGKIQDSLSSTASAL | 927-945 (C2) | H-2-Kd, H-2-Qa1 | H2-IAb | HLA-DRB1*04:01; HLA-DRB1*08:02 | HLA-A*02:01; HLA-A*02:03; HLA-A*68:02; HLA-B*07:02; HLA-B*08:01 |
| **C**PLQPELDSFKEELDKYFKNHTSPDVDLGDIS | | H-2-Kd | N/A | HLA-DPA1*02:01/DPB1*05:01; HLA-DRB1*04:01; HLA-DRB1*04:05; HLA-DRB1*13:02; HLA-DRB3*02:02 | HLA-A*01:01; HLA-A*23:01; HLA-A*24:02; HLA-A*26:01; HLA-A*30:01; HLA-A*30:02; HLA-B*08:01; HLA-B*35:01; HLA-B*44:03; HLA-B*53:01 |
| CVPAQEKNFTTAPAICHDGKAHFPREGVFVSNGTHWF | 1068-1103 (E2) | H-2-Db, H-2-Kb, H-2-Kd, H-2-Qa1, | H2-IAb | HLA-DRB1*03:01; HLA-DRB1*13:02; HLA-DRB3*01:01; HLA-DRB3*02:02; HLA-DRB5*01:01 | HLA-A*02:06; HLA-A*23:01; HLA-A*24:02; HLA-A*26:01; HLA-A*30:01; HLA-A*30:02; HLA-B*07:01; HLA-B*15:01; HLA-B*35:01; HLA-B*53:01; HLA-B*57:01; HLA-B*58:01 |
| CMTSCCSCLKGCCSCGSCCKFDEDDSEPVLKGV | 1236-1267 (F2) | H-2-Kk,H-2-Qa2 | N/A | N/A | HLA-A*68:02; HLA-B*40:01; HLA-B*44:02; HLA-B*44:03; HLA-B*51:01 |
